# Synthesis and Characterization of a Molecularly Imprinted Polymer of Spermidine and the Exploration of Its Molecular Recognition Properties

**DOI:** 10.3390/polym10121389

**Published:** 2018-12-14

**Authors:** Yu-Jie Huang, Rui Chang, Qiu-Jin Zhu

**Affiliations:** 1 School of Liquor and Food Engineering, Guizhou University, Guiyang 550025, China; loveyujie89@126.com (Y.-J.H.); guishanren@yeah.net (R.C.); 2 College of Food Safety, Guizhou Medical University, Guiyang 550025, China; 3 Key Laboratory of Agricultural and Animal Products Store and Processing of Guizhou Province, Guiyang, 550025, China

**Keywords:** spermidine, molecular imprint, polymerization, detection, density function theory

## Abstract

Spermidine is a functional ingredient that can extend the lifespan of many foods and indicate meat safety. However, its synthesis and enrichment is expensive and complex. To develop an effective separation material that can offer highly selective recognition of spermidine, we first applied non-covalent molecular imprinting technology using methacrylic acid as a functional monomer, azobisisobutyronitrile as an initiator, and ethylene glycol dimethacrylate as a cross-linker. The adsorption properties of the polymers were analyzed using the Scatchard equation, the Lagergren kinetic equation, and the static distribution coefficient. The optimal polymerization molar ratio of the template molecule spermidine to the functional monomer was 1:4, the maximum adsorption amount was 97.75 μmol/g, and the adsorption equilibrium time was 300 min. The selective experiment showed that the interfering substances tyramine and histamine had selectivity factor α values of 2.01 and 1.78, respectively, indicating that the prepared polymer had good spermidine recognition ability. The density function theory calculations showed that the hydrogen bond strength, steric effect, and product energy caused adsorption and separation differences among the different imprinted polymer complexes.

## 1. Introduction

The biological activity of functional food ingredients can involve cellular metabolism. The discovery of cell autophagy, which received the 2016 Nobel Prize in Physiology or Medicine, has extended human life [1], and our daily living is associated with numerous functional food ingredients that regulate cell autophagy levels [2]. For example, spermidine, which is widespread in soybeans, matured cheeses, and whole grains, plays roles in various life activities, and affects cell growth [3,4], demonstrating potential applications in the biological field [5]. However, spermidine can accumulate in contaminated aquatic and meat products, and pose a strong subacute toxicity when present in high amounts [6]. For example, spermidine and nitrite can produce carcinogens such as *N*-nitrosopiperidine in high-temperature-processed raw meat [7]. In general, spermidine is prepared by chemical synthesis and detected through electrochemistry, spectroscopy [8] and chromatography [9]; however, these methods have the disadvantages of high cost and bad test results. Herein, we tried to enrich and identify spermidine by molecular imprinting technology with a simple polymerization method.

The principle of molecular imprinting technology is to create artificial recognition sites in polymeric matrices that are complementary to the template in terms of size, shape, and spatial arrangement of the functional groups. The recognition ability depends on the presence of covalent/non-covalent bond interactions and the special geometrical shape of the cavity [10]. Molecularly imprinted polymers (MIPs) are resistant to mechanical, acid, and alkali damage, and have therefore been widely used in high value-added substance enrichment [11], chemical processes [12], biosensing [13,14], surface functional coatings [15], and environmental detection [16,17]. MIPs can be prepared by covalent (pre-assembly method) and non-covalent (self-assembly method) methods, which involve different ways of combining template molecules and functional monomers. Covalent bonding produces reversible covalent bonds between monomers and template molecules. In non-covalent methods, polymers and imprinted molecules are arranged spontaneously and orderly via molecular interaction forces such as hydrogen bonds, metal chelation, electrostatic attraction, and hydrophobic effects [18]. Density functional theory (DFT) [19], atoms in molecules (AIM) [20], and reduced density gradient (RDG) function are all based on the electron density of atoms, and are widely used to analyze interactions between host and guest systems [21]. 

Currently, little research has been done on spermidine-imprinted polymers. Some researchers have prepared a molecularly imprinted fluorescent chemical sensor for the detection of spermidine by quinoline modified *β*-cyclodextrin and acrylamide [22,23]. Here, we used another method to prepare a spermidine-imprinted polymer, and in order to verify the results of our previous theory calculations results on spermidine MIPs.

In our previous work, the optimal ratio of the spermidine–methacrylic acid complex was predicted to be 1:5 using the binding energy and hydrogen bonding calculation [24]. However, the intermolecular weak interaction analysis of the complex in our previous work was insufficient. Therefore, in this study, we prepared a spermidine molecularly imprinted polymer using the precipitation polymerization method. The appropriate polymerization ratio of the functional monomer to the template molecule was chosen from our previous theory calculation results, and we used spermidine-to-methacrylic acid (SPD–MAA) polymerization ratios of 1:3, 1:4, and 1:5. 

## 2. Materials and Methods 

### 2.1. Materials and Reagents

The following substances were used: spermidine (purity ≥ 99%); tyramine (purity 99%); histamine (purity ≥ 98%); methacrylic acid (purity 99%); ethylene glycol dimethacrylate (EGDMA, purity 98%, Sigma-Aldrich co., St. Louis, MO, USA), azobisisobutyronitrile (AIBN, analytical grade, Chengdu Gracia Chemical Technology Co., Ltd., Chengdu, China); carbon tetrachloride (analytical grade, Tianjin Kemi Europe Chemical Reagent Co., Ltd., Tianjin, China); and nitrogen (Guizhou Yongsheng Industrial Gas Company, Guizhou, China). 

### 2.2. Instruments and Equipment

The following instruments and equipment were used: a WYH-2 constant temperature water bath oscillator (Jintan Jingda Instrument Manufacturing Co., Ltd., Jintan, China); a HH-W420 constant temperature water bath (Xingtai Runlian Machinery Co., Ltd., Xingtai, China); an SB-100D ultrasonic cleaning machine (Ningbo New Chi Biotechnology Co., Ltd., Ningbo, China); an FA2004B electronic analytical balance (Shanghai Rong Research Instrument Co., Ltd., Shanghai, China); a Spectra Max 190 microplate reader (Molecular Devices, Corporation, Sunnyval, CA, USA); AP-01P vacuum pump (Tianjin Ott Saens Instrument Co., Ltd., Tianjin, China); and a Guizhou University High Performance Computing Platform. 96-well quartz microplates (Hellma, NY, USA).

### 2.3. Method

#### 2.3.1. Synthesis of Spermidine MIPs

In accordance with our previous theoretical research work, the optimal molar ratio of the selected functional monomer to the template molecules was achieved through the DFT of the calculation chemistry. The molecular structural model of spermidine combined with different monomers (methacrylic acid, acrylamide, acrylic acid and so on) was fully considered, as it is important to choose the appropriate functional monomer during the preparation of MIPs. The predicted optimal ratio was 1:5. Methacrylic acid can form hydrogen bonds with amides, esters, and carboxyl compounds, and it forms ionic bonds with amines [25]. Methyl has an electron donor effect and an electron hyperconjugation effect, and the double bond of olefin is highly vulnerable to electrophilic attack. Thus, the methacrylic group is enriched with more electrons than the two other functional monomers, and a stronger hydrogen bond with more nucleophilic spermidine is produced. This result is consistent with that of Kerstin Golker [26], who conducted a molecular dynamic simulation of the interaction of functional monomers with template molecules. 

Here, MIPs were prepared through non-covalent polymerization. The SPD–MAA polymerization ratios used were 1:3, 1:4, and 1:5. First, 217.5 mg of the spermidine standard was accurately weighed, placed in a 250-mL triangular conical flask, and added to 150 mL of carbon tetrachloride solution for dissolution. Then, methacrylic acid, a cross-linking agent (EGDMA), and an initiator (AIBN) were mixed in appropriate molar ratios and amounts (Table 1). Next, the mixture was stirred until dissolution, and bubbles were discharged by ultrasound treatment [27]. Afterwards, the mixture was purged with nitrogen for 20 min to completely remove the oxygen. Then, the triangular flask was sealed with a thin film, placed in a water bath at 55 °C, and heated for 24 h until the reaction was complete.

To compare the structural differences among the imprinted polymers, we prepared non-molecularly imprinted polymers (NIPs) by replacing only the template molecule spermidine with the solvent carbon tetrachloride in proportion and retaining the same preparation conditions as those for MIPs. 

#### 2.3.2. Polymer Elution Treatment

A triangular flask was removed once the reaction was complete. The surface of a Buchner funnel was lined with filter paper, and the polymer and the solution were poured into the funnel with a vacuum circulating pump for suction filtration. Methanol was added to elute the incompletely reacted chemicals and impurities attached to the surface of the MIP during filtration to prevent them from affecting subsequent reactions [28]. 

After the suction filtration was complete, the polymer was freeze-dried at −4 °C under vacuum. Afterwards, the powdered polymer was placed in a Soxhlet extractor in a water bath at constant temperature, and the temperature was set to 75 °C to elute the template molecules. Elution was carried out using a standard acetic acid–methanol solution (*V*/*V* = 1:9), and the Elution was finished until spermidine cannot be detected in the supernatant.

#### 2.3.3. Spermidine Standard Curve Plot

**Figure 1:**
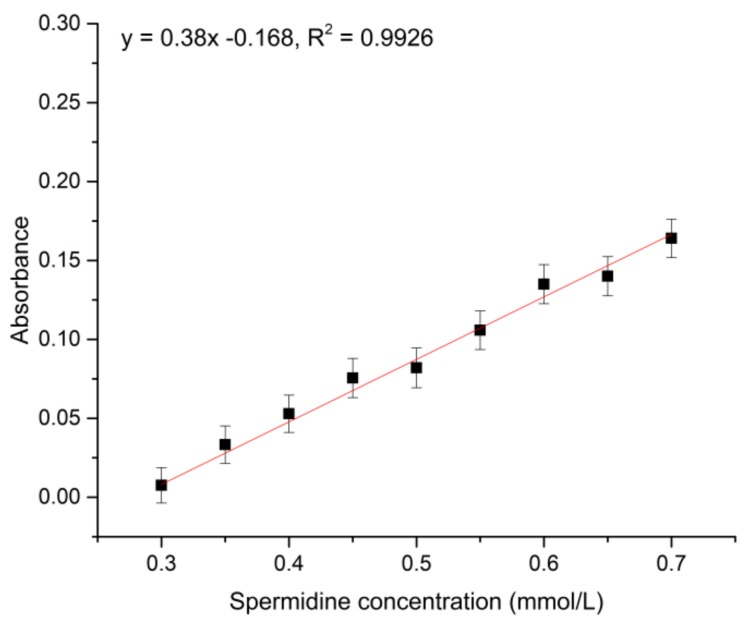
Standard spermidine curve.

A standard spermidine sample (72.6 mg) was accurately weighed and combined with carbon tetrachloride as a solvent in a 50 mL volumetric flask to prepare a 10 mmol/L standard sample mother liquor. For each different concentration of the standard solution (range of 0.3–0.65 mmol/L), 200 μL was placed in a 96-well plate and subjected to 260 nm UV scanning in a microplate reader. Carbon tetrachloride solvent was used as a blank control, and the test was conducted in parallel three times. The results are shown in Figure 1.

We selected standard spermidine to exclude impurity interference. The linear relationship of the standard curve was as follows: *y* = 0.38*x* − 0.1068, *R*² = 0.9926.

#### 2.3.4. Spermidine MIPs Adsorption Kinetics and Determination of Adsorption Equilibrium Time 

MIPs rely on interactions between molecules to achieve the dynamic adsorption and release of target molecules. The most important parameter characterizing the adsorption strength is the adsorption equilibrium time. This may refer to the reference time needed to find the adsorption capacity of MIPs in saturated adsorption rather than the time when the adsorption amount peaks [29]. Initially, a certain amount of standard spermidine was weighed, and a 0.475 mmol/L adsorption solution was prepared with carbon tetrachloride. Next, 20 mg (±0.5 mg) of MIPs was weighed in a 10 mL centrifuge tube, 5 mL adsorption solution was added to each tube, and the adsorption time was set to 10, 20, 30, 60, 90, 120, 150, 180, 240, 300, 360, or 480 min. When adsorption was complete, the samples were centrifuged for 5 min at 12,000 r/min. Afterwards, the supernatant was filtered through an 0.22 μm oily organic membrane, 200 μL was drawn from the filtrate, and parallel sampling was conducted thrice. Absorbance was measured at 260 nm with the microplate reader. The remaining spermidine solution concentration was calculated in accordance with the standard curve. Equation (1) was used to calculate the concentration of MIPs during the determination of the adsorption time [30,31]:

(1) $$Q=(C_{0}-C_{n})\times \frac{V}{m}$$

where *Q* is the adsorption capacity (mg/g); *C*_0_ is the initial concentration of the adsorption solution (mmol/L); *C*_n_ is the remaining concentration of the adsorbate (mmol/L) (*n* = 1, 2, 3,...); *V* is the volume of the added adsorption solution (mL); and *m* is the MIPs mass (g). The kinetic adsorption equilibrium time and saturation adsorption time were obtained by plotting the adsorption time and capacity. To calculate the theoretical adsorption value, we first fitted the data with the kinetic equation. 

#### 2.3.5. Spermidine MIPs Adsorption Kinetics and Adsorption Capacity Determination

A series of adsorption solutions with concentrations of 0.450, 0.475, 0.500, 0.525, 0.550, 0.575, and 0.600 mmol/L spermidine standard were prepared with carbon tetrachloride as the solvent. Then, 20 mg of MIP standard was added to eight centrifuge tubes (volume = 10 mL), and 5 mL of different concentrations of the above-mentioned adsorption solution was added. Next, the tubes were sealed and placed in a shaker at a constant temperature of 25 °C, and the shock adsorption time was set, using the MIP saturated adsorption time as a reference. When the shock adsorption had finished, a certain amount of each sample was obtained at the adsorption equilibrium time and centrifuged at 12,000 r/min for 5 min. The sample was then filtered with a 0.22 m organic phase filter, and 200 μL of the filtrate was drawn three times in parallel. The test absorbance was obtained at 260 nm in a microplate reader. Adsorption capacity (*Q*) of MIPs/NIPs was calculated using Equation (1). 

The Scatchard equation is often used to examine and quantitatively analyze receptor and ligand binding. Like the process of enzyme and receptor binding, the adsorption process of MIPs also involves dissociation equilibrium. The dissociation constant and the maximum apparent binding capacity can be estimated by the results of the saturation adsorption experiment to allow the quantitative analysis of the binding process. A Scatchard analysis was conducted to further investigate the binding properties of the MIPs [32], which were characterized by binding parameters determined by the Scatchard equation in accordance with Equation (2):

(2) $$\frac{Q_{e}}{C_{e}}=\frac{\left(Q_{\max}-Q_{e}\right)}{K_{d}}$$

where *Q*_e_ is the amount of adsorption of equilibrium MIPs; *C*_e_ is the concentration of spermidine in the solution at equilibrium; *Q*_max_ is the maximum apparent binding capacity; and *K*_d_ is the equilibrium dissociation constant of the MIP binding site. Then, we plotted a linear fitting curve with *Q*_e_/*C*_e_ against *Q*_e_ to obtain the maximum apparent binding and the equilibrium dissociation constant.

#### 2.3.6. Selective Adsorption of Spermidine MIPs 

The spatial induced conjunction hypothesis can explain the recognition of functional monomers by MIPs based on certain space similarity [33]. Tyramine and histamine, which have geometric structures analogous to that of spermidine, were selected as interfering substances to examine the adsorption selectivity of the prepared imprinted polymer. The two interfering substances were configured into a 0.6 mmol/L solution with carbon tetrachloride, and then 5 mL of the solution was placed into a 10 mL centrifuge tube. The tubes were sealed after the addition of 20 mg of spermidine MIP, before being placed in a 25 °C shaker. The shock adsorption time was the same as the previous absorption equilibrium time. The adsorption levels at this concentration were calculated using Equation (1). To provide a further realistic comparison, we applied the same treatment to the NIPs. Molecular imprinting is an efficient separation technique. The selective adsorption process of imprinted and non-imprinted polymers is similar to that of chromatographic separation. The selective adsorption capacity is therefore characterized by the static partition coefficient K and the selectivity factor α. *K* = *Q*/*C*_e_, where *Q* represents the concentration of the adsorbed liquid on the MIPs (μmol/g) and *C*_e_ is the solution concentration after adsorption equilibrium (mmol/L). Moreover, α = *K*_i_/*K*_j_, where i is the template molecule and j is the interfering substance when *i* = *j* and *α* = 1 [34]. 

#### 2.3.7. Intermolecular Interaction Analysis 

To understand the more detailed inner mechanism of the MIPs recognition process, we applied the atom in molecules theory and reduced the density gradient function to explore the weak intermolecular interactions based on the three different ratio complex structures obtained from our previous work (optimized at B3LYP/6-311 G(d,p) theory level ). The calculation level was M062X/6-311+G(d,p) at the water phase, and the solvent effect was considered using the SMD implicit solvent mode. We also performed the intermolecular interaction energy decomposition of the Spermidine-MAA complex at different ratios. and all this part was performed on the free download Multiwfn 3.6 packages [35]. 

Based on thermodynamic principles, the recognition process tends to forms complex products with the lowest energy levels. Therefore, to gain insight into the probable reasons for the different selectivity abilities of the MIPs (ratio 1:4), the total energy levels of three spermidine, tyramine, histamine, and methacrylic acid complexes (also with ratios of 1:4) were calculated at the M062X/6-311++G(d,p) level, and the optimization level was B3LYP/6-311+G(d,p). The energy level was corrected by the basis set superposition error (BSSE) [36]. All of the quantum chemical calculations throughout this work was completed on the Gaussian 16, revision A. 03 packages [37] that are supported by the Guizhou University High performance computing platform.

## 3. Results and Discussion

### 3.1. Adsorption Equilibrium Time of Spermidine MIPs

**Figure 2:**
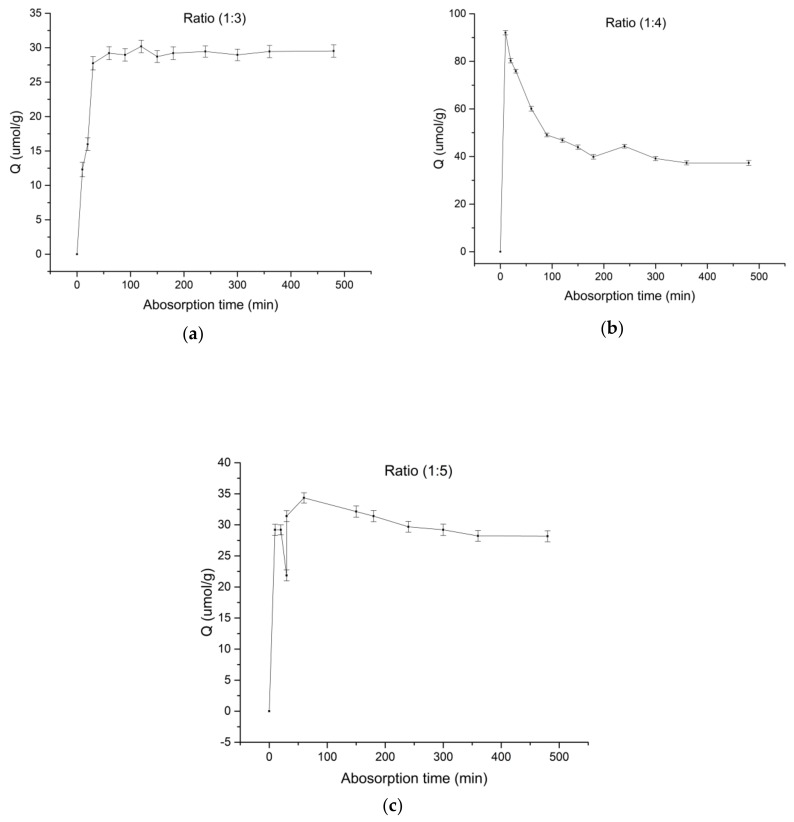
Curves of the binding dynamics of the three MIPs. (**a**) Ratio = 1:3, (**b**) ratio = 1:4, (**c**) ratio = 1:5 (at 25 °C, constant temperature shaker).

In accordance with Section 2.3.4, three molar ratios of the imprinted polymers were selected to determine the adsorption equilibrium time, the results of which are shown in Figure 2. The a, b, and c ratios of SPD–MAA are 1:3, 1:4, and 1:5, respectively.

### 3.2. Adsorption Equilibrium Time of Spermidine MIPs

Figure 2 shows that the adsorption capacity increased with time when the SPD–MAA ratio was 1:3; adsorption equilibrium was reached at about 300 min. However, the adsorption capacity of the MIPs did not change substantially. Thus, at time *t* = 300 min, the MIPs reached equilibrium adsorption. When the SPD–MAA ratio was 1:4, the adsorption capacity of the MIPs did not increase with the adsorption time, but first reached maximum absorption at 10 min, which was still not the equilibrium time, and finally reached maximum absorption at 150 min. At this point, the template molecule spermidine adsorbed by the MIPs was gradually released until the system reached equilibrium at 200 min. This phenomenon shows that the previous adsorption was non-specific because the spermidine molecules attaches to the surfaces of the MIPs and cannot form stable complexes with MIPs’ highly selective recognition site; thus, it was easy for spermidine to be released over time. The MIPs possibly had a rough surface and a large specific surface area, which made the adsorption process dynamic. Therefore, non-specific adsorption could affect selective adsorption capacity. When the SPD–MAA ratio was 1:5, the adsorption of the polymer generally increased over time. Within 20–120 min, non-specific adsorption occurred and then gradually disappeared. Compared to the saturated adsorption capacity, the maximum equilibrium adsorption was 39.1 μmol/g at 1:4, followed by 29.7 μmol/g at 1:5, and the lowest value of 28.9 μmol/g was obtained at 1:3. However, the maximum equilibrium adsorption was the saturated adsorption amount reached by the MIPs at this concentration, rather than the maximum apparent adsorption amount of MIPs. To explore the MIPs’ adsorption features further, we adopted the adsorption kinetic equation for fitting analysis. The equations commonly used to simulate the adsorption formula are the Lagergren first-order kinetic model and the Lagergren pseudo-second-order kinetic model [38,39], as follows:

(3) $$\frac{dQ_{t}}{dt}=k_{1}(Q_{e}-Q_{t})$$

In Equation (3), *Q*_t_ is the amount of adsorption of MIP (μmol/g) at time t, *Q*_e_ is the equilibrium time adsorption capacity, and *K*_1_ is the quasi-first-order kinetic equation rate constant (min^−1^). Alignment was conducted in the first-order kinetic equation for linearization to obtain Equation (4):

(4) $$\log(Q_{e}-Q_{t})=\log Q_{e}-\frac{k_{1}t}{2.303}$$

where C is a constant, and the time variable t is plotted to fit K_1_. Meanwhile, the Lagergren quasi-second-order kinetic model is as shown in Equation (5):

(5) $$\frac{dQ_{t}}{dt}=k_{2}{\left(Q_{e}-Q_{t}\right)}^{2}$$

where Q_*t*_ is the amount of adsorption of the MIPs (μmol/g) at time *t*; Q_e_ is the equilibrium time adsorption capacity; and K_2_ is the quasi-second-order kinetic equation rate constant (g/min^−1^·mg^−1^). Alignment of the second-order kinetic equation for linearization is shown in Equation (6):

(6) $$\frac{t}{Q_{t}}=\frac{1}{k_{2}Q_{e}^{2}}+\frac{t}{Q_{e}}.$$

The plot of *t*/*Q*_t_ can be fitted to obtain *K*_2_ and *Q*_e_. The two equations were solved using the data in Figure 2, and the correlation coefficients obtained are revealed in Table 2. On the basis of the correlation coefficient *R*^2^, we found that when the data were substituted into the quasi-first-order equation, the results were not ideal. Hence, a follow-up analysis was not carried out.

By substituting the data of each adsorption time into the quasi-second-order kinetic equation, we obtained the fitting result to the time t/*Q*_t_ (min) and processed the fitting curve data, as shown in Table 3.

Table 2 and Table 3 show the adsorption process of the MIPs. The quasi-first-order kinetic equation can be used in the initial stage of the adsorption process. From Table 3, the quasi-second-order kinetic equation was more suitable for use over the entire adsorption process. The results showed that the adsorption process of the MIPs can be divided into two steps. As suggested by the changes in *R*^2^, the adsorption of spermidine during molecular imprinting is the first step, which is fast and unstable. Hence, at 1:4 and 1:5, the MIP adsorption capacity surged within a short period and produced a high adsorption peak. However, the adsorbed spermidine could easily fall from the surface layer as adsorption was only carried out on the surface. Thus, the amount of adsorption gradually decreased. This phenomenon has also been found in many other studies [40,41]. 

The second step was specific adsorption. During adsorption, some of the spermidine molecules were affected by non-covalent bonds of the MIPs holes, and then gradually transferred from the surface to the inner holes of the MIPs, finally forming stable binding (spermidine’s highly selective binding with MIPs by hydrogen bonding). The adsorption of the second step was more stable than that of the first; thus, it was difficult for the combined spermidine molecules to detach from the holes. In conclusion, Lagergren quasi-second-order kinetics can thoroughly describe the process of the MIP adsorption of spermidine in solution.

### 3.3. Adsorption Properties of Three Spermidine MIPs Ratios 

**Figure 3:**
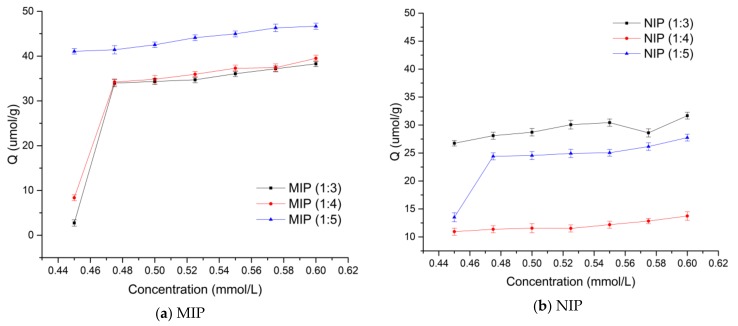
Proportion of the MIP isothermal adsorption curve: (**a**) MIPs, (**b**) non-molecularly imprinted polymers (NIPs) (at 25 °C, constant temperature shaker).

The adsorption properties of the MIPs and NIPs at the three different polymerization ratios were investigated through an equilibrium adsorption experiment (Figure 3).

As shown in Figure 3, in the range of 0.45–0.60 mmol/L, as the concentration of the adsorbate increased, the amount of adsorption on the MIPs/NIPs also increased. The adsorption capacity of MIPs at a given concentration was greater than the amount of adsorption of NIPs. The adsorption process involves two steps: the saturated adsorption of polymers, which is achieved when the spermidine molecules have finished highly selective adsorption. At the same concentration, the amount of adsorption of the MIPs was also found to be larger than that of the NIPs, which indicates that there were more highly selective recognition and adsorption sites for spermidine in MIPs than NIPs. 

**Figure 4:**
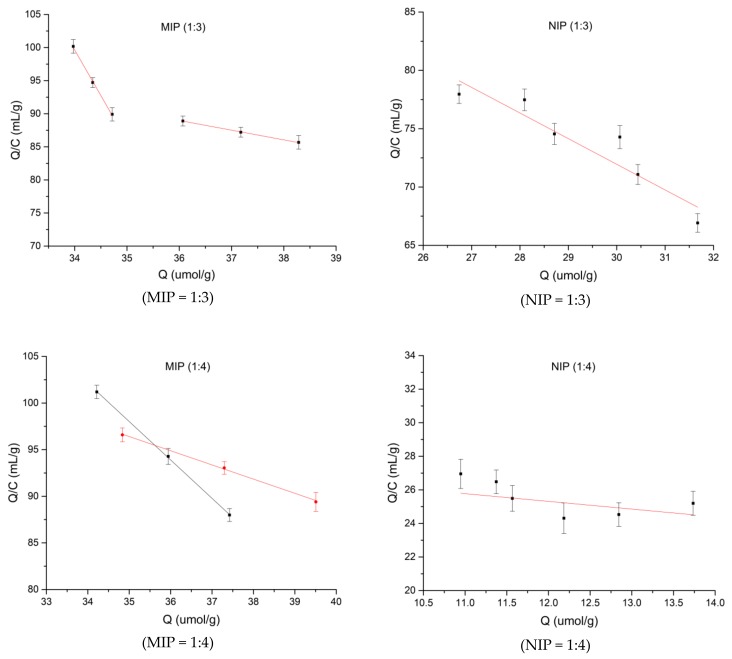

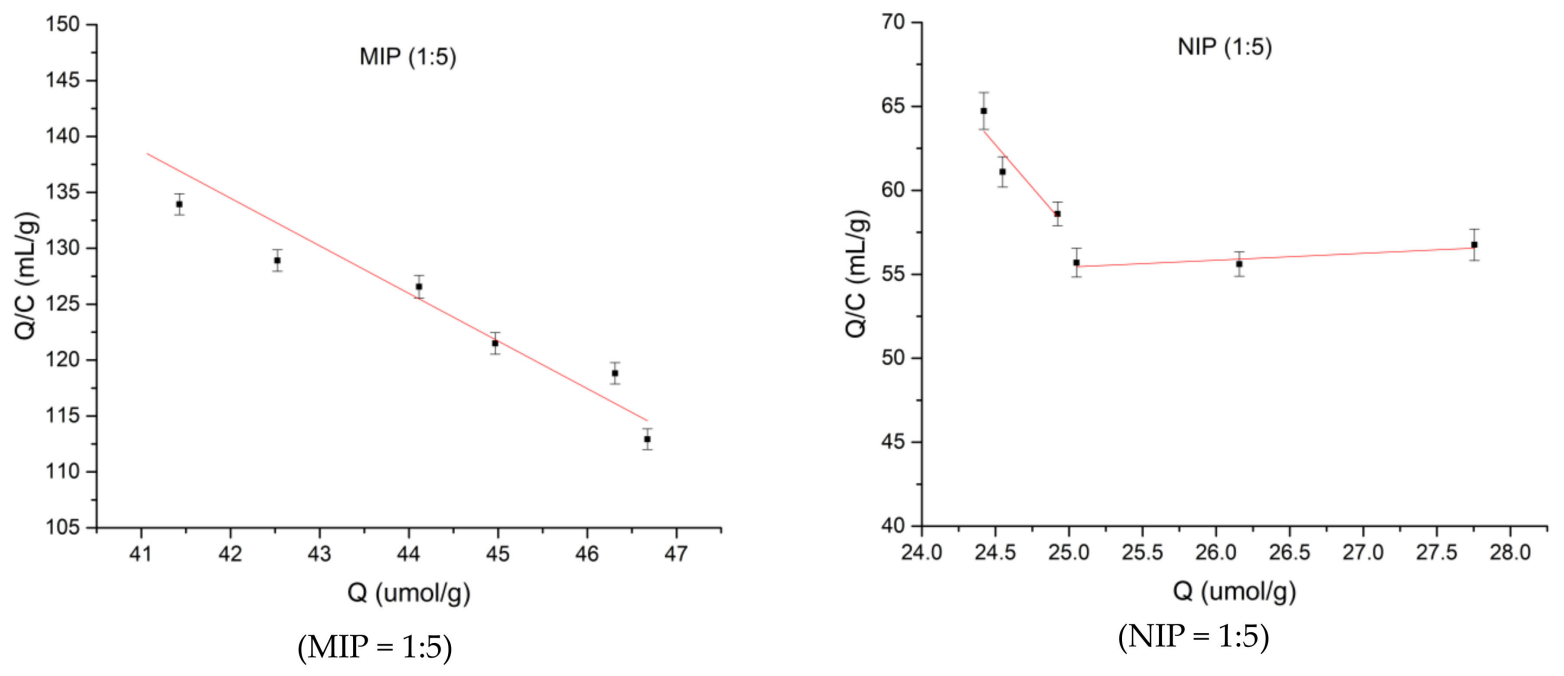
MIP/NIP Scatchard fitting figure. Note: MIP and NIP from left to right (at 25 °C, constant temperature shaker).

In accordance with Equation (3), the adsorption amounts and concentrations of the MIPs and NIPs obtained in Section 2.3.5 were calculated. The data from absorption are further processed with Scatchard analysis (see Figure 4).

Figure 4 shows the MIP/NIP Scatchard fitting chart of various polymerization ratios. The fitting data indicates that MIP at the ratio of the template to the functional monomer was 1:3, the high binding site equation was *Q*/*C* = –1.45x + 141.45 (*R*^2^ = 0.9992). The low binding site equation was *Q*/*C* = –13.7*x* + 567.83 (*R*^2^ = 0.9986). The dissociation constants were 0.69 μmol/L and 0.073 μmol/L, respectively. The maximum apparent adsorption amounts were 97.55 μmol/g and 41.45 μmol/g. The non-imprinted polymer (1:3) fitting result was *Q*/*C* = –1.71x + 122.01 (*R*^2^ = 0.236). The equilibrium dissociation constant was 0.58 μmol/L and *Q* = 71.35 μmol/g. When the template to functional monomer ratio was 1:4, the fitting results for the MIP polymer were the high binding site equation was *Q*/*C* = –1.53*x* + 150.17 (*R*^2^ = 0.9985) and the low binding site equation was *Q*/*C* = –4.11*x* + 242.1 (*R*^2^ = 0.9997). The equilibrium dissociation constants were 0.65 μmol/L and 0.24 μmol/L, respectively. The maximum apparent adsorption levels were 98.15 μmol/g and 58.91 μmol/g, respectively. At the 1:4 ratio, the non-imprinted polymer was fitted to *Q*/*C* = –0.52*x* + 31.62 (*R*^2^ = 0.2164). The dissociation constant was 1.90 μmol/L, and the maximum apparent adsorption capacity was *Q* = 60.04 μmol/g. MIP at ratio of 1:3 and 1:4 had two kinds of binding sites, high binding sites and low binding sites [42], because in non-covalent polymerization, different types of non-covalent bonds of the template and monomer were combined to form different complexes and adsorption sites [43]. With the 1:5 ratio, after fitting, a straight line was produced: *Q*/*C* = –4.291*x* + 314.74 (*R*^2^ = 0.9985). The equilibrium dissociation constant was 0.233 μmol/L and the maximum apparent adsorption capacity was 73.35 μmol/g. This shows that only one binding site appeared when the ratio of template to functional monomer was 1:5, For the non-imprinted polymers synthesized at a 1:5 ratio, *Q*/*C* = –10.99*x* + 332.21 (*R*^2^ = 0.8618), the equilibrium dissociation constant was 0.091 μmol/L, and the maximum apparent adsorption capacity was 30.22 μmol/g. The largest adsorption capacity of 98.15 μmol/g was detected at 1:4, followed by 97.55 μmol/g at 1:3, and 73.35 μmol/g at 1:5. The template and the functional monomer at the polymerization ratios of 1:3 and 1:4 formed two different binding sites that tend to be specifically adsorbed spermidine by non-covalent bonds. When the polymerization ratio was 1:5, only a single adsorption site was attained during the adsorption process. For all three non-imprinted polymers, the linear relationships were not good. The adsorption capacity of NIPs was less than that of the MIPs, with 71.35 μmol/g at the ratio of 1:3, 60.04 μmol/g with the 1:4 ratio, and 30.22 μmol/g with the 1:5 ratio. This was because, on the one hand, the polymer could not form voids and binding sites for the selective adsorption of spermidine in the absence of template molecules. On the other hand, the the NIPs can also adsorb spermidine molecules through intermolecular interactions such as Van der Waals forces.

From the above figures, we can conclude that the non-covalent interactions derived from the template and function monomer can form two different types of binding sites. The produced Scatchard plots of the MIPs are non-linear. Two straight lines can be drawn through the points, which indicates that the affinities of the binding sites in the MIPs are heterogeneous and can be approximated by two dissociation constants corresponding to the high-and low-affinity binding sites that are typical in Scatchard analyses [44]. The heterogeneity of the recognition sites inside the MIPs at the ratios of 1:3 and 1:4 indicates that two kinds of recognition sites exist, and they act independently of each other. Furthermore, the recognition sites in the NIPs were also shown to be homogeneous since the data could be fitted with a single straight line. The interactions between spermidine and the NIPs mainly arose from non-selective interactions, such as van der Waals interactions [45,46]. 

### 3.4. Selective Adsorption Test of Spermidine MIPs 

The selective adsorption capacity test was then performed in accordance with Section 2.3.6. Two common biogenic amines (tyramine and histamine) were chosen for the experiments. The selective adsorption capacities of the MIPs and NIPs were characterized by the static partition coefficient K and the selectivity factor *α*. We used the equation *K* = *Q*/*C*_e_, where *Q* represents the concentration of the adsorbate on the MIPs (μmol/g) and *C*_e_ is the solution concentration after adsorption equilibrium (mmol/L). We also used the formula *α* = *K*_i_/*K*_j_, where i is the template molecule and j is the interfering substance. The results for *i* = *j* and *α* = 1 are shown in Table 4.

### 3.5. Intermolecular Interaction Analysis of MIPs

The AIM theory is based on the electron density of molecular systems and is often used to reflect the strength and properties of intermolecular hydrogen bonds. Additionally, the bond critical points (BCP) are related to the pathways of hydrogen bond donors and receptors [47]. The general criteria for an intermolecular hydrogen bond are the presence of a donor and receptor within 3.9 angstroms and an angle of less than 90 degrees [48]. The BCP search and intramolecular hydrogen bond topological analysis of SPD–MAA at different ratios are shown in Table 5. Note that ρ represents the electron density; *∇*^2^_ρ_ represents the Laplacian of electron density; *G* represents the Lagrangian kinetic energy; *H* represents the Hamiltonian kinetic energy; and *V* represents the potential energy density. 

Rozas et al. divided hydrogen bonds into three types: weak (*E*_HB_ < 12.0 kcal/mol, ∇^2^_ρ_ > 0, *G*_BCP_ + *V*_BCP_ > 0), medium (12.0 < *E*_HB_ < 24.0 kcal/mol, ∇^2^_ρ_ > 0, *G*_BCP_ + *V*_BCP_ < 0), and strong (*E*_HB_ > 24.0 kcal/mol, ∇^2^_ρ_ < 0, *G*_BCP_ + *V*_BCP_ < 0 ) [49]. According to the classification criteria, the hydrogen bonds formed in SPD–MAA complex were all weak hydrogen bonds (*E*_HB_ < 12.0 kcal/mol, ∇^2^_ρ_ > 0, *G*_BCP_ + *V*_BCP_ > 0). Among them, we found four BCPs at the 1:3 complex, six at the 1:4 complex, and seven at the 1:5 complex. By comparing each of the topological parameters, the searched hydrogen bond strength between the molecules at the 1:4 ratio was slightly larger than that at the 1:5 ratio. 

The non-bond interactions in the molecular force field include electrostatic and van der Waals, and the van der Waals can be divided into repulsions that act as repulsive and dispersions that act as attractors [50]. In order to understand the detailed contribution of molecular interaction force between spermidine and every functional monomer, we used the Multiwfn software to make the energy decomposition based on the molecular force field. Considering the Amber force field was better for organic molecules [51], we finished the calculation at Amber force field. The atomic charge was calculated at the B3LYP/6-311+G** level; the results are listed in Table 6. 

From Table 6, we can see that the electrostatic and dispersion force contributed to the binding process, while the repulsion force was averse to it [52]. For ratio 1:3, the electrostatic and dispersion values were the lowest, indicating that it is difficult to recognize and attract spermidine molecules; this was consistent with our maximum adsorption capacity result. The electrostatic interaction was related to hydrogen bond formation. The AIM theory was based on atomic electron density, which was also related to the nature of hydrogen bonding. Table 6 shows that the electrostatic values for ratio 1:4 was lower than ratio 1:5, this was consistent with the AIM theory bond critical point search results of inter-molecular hydrogen bond numbers at ratio 1:4 and ratio 1:5 was six and seven, respectively. As the dispersion was mainly related to the solvation energy, and generally less critical [53], the recognition process was mainly dominated by the repulsion and electrostatic force. It was obvious that ratio 1:5 formed a more repulsive force than 1:4, which may be the reason for the lower adsorption capability than ratio 1:4 in the actual experiment. 

The reduced density gradient (RDG), which is based on the electron density union with sign (λ2) functions, is a good tool to show weak non-covalent interaction regions. The general equation is as follows [54]:

(7) $$\mathrm{RDG}\;(r)=\frac{1}{{2(3\pi }^{2}{)}^{1/3}}\frac{\left|\nabla \rho (r)\right|}{{\rho (r)}^{4/3}}.$$

**Figure 5:**
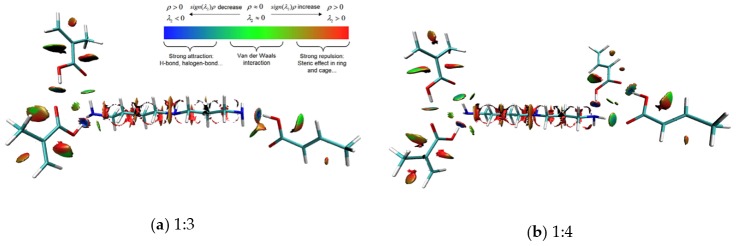

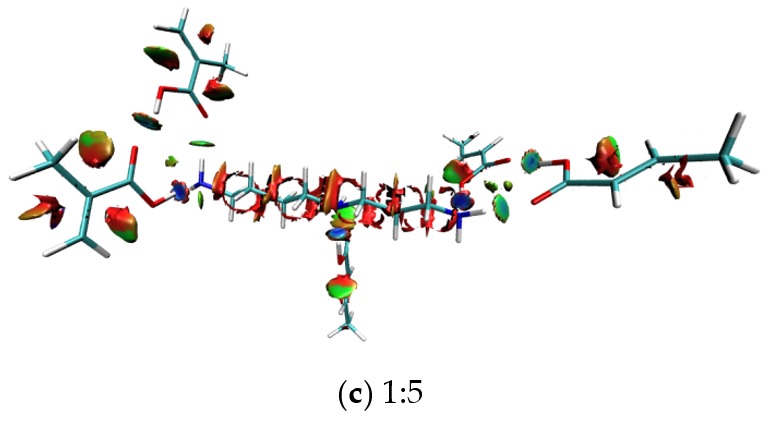
Three-dimensional colored isosurfaces of the three ratios of SPD–MAA complex non-covalent interactions: (**a**) ratio 1:3; (**b**) ratio 1:4; and (**c**) ratio 1:5.

When mapping *ρ*(r) against the sign (*λ*_2_), there existed a three-dimensional isosurface distribution map of intermolecular weak interactions. Green represents van der Waals interactions, red represents the space repulsive and steric effect, and blue represents the electrostatic interactions and hydrogen bonds. Three-dimensional colored isosurfaces of the non-covalent interactions at the three ratios of SPD–MAA complexes are shown in Figure 5.

As Figure 5 shows, the intermolecular interactions of the SPD–MAA complex systems were donated by the electrostatic and spatial repulsive forces. The differences in the electrostatic interactions of these three are obvious. The 1:5 complex showed the most blue areas, which indicates that weak hydrogen bonds play an important role in recognition. However, the 1:5 ratio complex also showed more red regions as a result of producing more steric and space repulsion than the other complexes, which may prevent additional spermidine molecules binding to the cavities of the MIPs; this is consistent with the results of the intermolecular force energy decomposition and adsorption experiment. The hydrogen bond strength and steric effect differences may explain why the adsorption capacity of the prepared SPD–MAA polymers (1:4) was actually much better than that of the complexes with the 1:5 ratio.

The calculated total energy of the two interfering substances (tyramine, histamine) and the methacrylic acid 1:4 complex is shown in Table 7. Energy production satisfied the order with *E*_spermidine-MAA_ < *E*_tyramine-MAA_ < *E*_histamin-MAA_, which is consistent with the highly selective adsorption test of spermidine MIPs. In terms of the molecular structure, histamine had the same number of amino groups (binding sites) as spermidine, while tyramine only had two likely binding sites, thus producing differences during the binding process. Therefore, the selective separation effects of the prepared polymers on histamine was not as good as with tyramine.

## 4. Conclusions

This study used spermidine as a template molecule and methacrylic acid as a functional monomer, and successfully synthesized MIPs with a highly selective recognition ability for spermidine and good anti-interference ability on tyramine and histamine through a precipitation polymerization method. The polymer prepared by this method had a small and uniform particle size and could be used without grinding and screening. The Lagergren model showed that the adsorption process was close to the quasi-second-order kinetic equation. The Scatchard equation-fitted adsorption process showed that the imprinted polymer at polymerization ratios of 1:3 and 1:4 MIPs produced both highly selective and non-specific affinity to spermidine molecules, while at a ratio of 1:5 MIPs, the main force present was highly selective adsorption. The maximum adsorption amount and the selectivity factor α revealed that the polymerization ratio of 1:4 was the best, the highest adsorption of spermidine was 97.75 μmol/g, and the selectivity factors for histamine and tyramine were 2.01 and 1.78, respectively—much higher than those of the non-imprinted polymers. The theory calculation analysis provided more molecular level details of the recognition interaction processes. The atom in the molecules, intermolecular interaction energy decomposition, and reduced density gradient function theory calculations found that the hydrogen bond strength and steric effect difference were the main reasons for the better adsorption capacity of the polymerization ratio at 1:4 than at 1:5 and 1:3. The difference of the functional groups at interfering substance was the main reason for various selection ability of MIPs. This work provides a reference for exploring the recognition characteristics of molecular imprinting polymers.

## Figures and Tables

**Table 1:** Proportion of molecularly imprinted polymer (MIP) synthetic material.

Template molecule: Spermidine (mg)	Functional monomer: Methacrylic acid (mL)	Molar ratio of Template molecule to functional monomer	Initiator AIBN (mg)	Cross-linker EGDMA (mg)
217.5	381.60	1:3	100.00	2.00
217.5	510.00	1:4	100.00	2.00
217.5	642.50	1:5	100.00	2.00

**Table 2:** Fitting correlation coefficients of the Lagergren pseudo-first-order kinetic model.

Ratio	*R* ^2^
1:3	0.3372
1:4	0.5662
1:5	0.0233

**Table 3:** Results of the Lagergren pseudo-second-order kinetic model.

Ratio	*Q*_e_ (μmol/g)	*Q*_e_ caq(μmol/g)	*K*_2_ (g/μmoL/min)	*R* ^2^	*Q*%
1:3	28.95	30.04	0.004804	0.9985	3.76
1:4	39.14	36.41	0.001422	0.9949	6.99
1:5	29.20	28.53	0.02328	0.9945	2.29

**Table 4:** Distribution coefficient K and selectivity factor α of MIPs/NIPs.

Substance	MIP (1:3)			NIP (1:3)		
	***Q* (μmol /g)**	***K*_i/j_ (mL/g)**	***α***	***Q* (μmol /g)**	***K*_i/j_ (mL/g)**	***α***
**Spermidine**	38.24	85.55	-	31.67	66.96	-
**Tyramine**	24.75	49.41	1.73	24.75	49.4	1.35
**Histamine**	26.73	54.21	1.58	26.11	52.69	1.27
**Substance**	**MIP (1:4)**			**NIP (1:4)**		
**Spermidine**	39.51	89.80	-	13.74	24.98	-
**Tyramine**	22.66	44.48	2.01	9.11	16.16	1.56
**Histamine**	25.09	50.22	1.78	10.5	18.81	1.34
**Substance**	**MIP (1:5)**			**NIP (1:5)**		
**Spermidine**	46.67	113.01	-	27.75	56.74	-
**Tyramine**	30.36	63.44	1.78	22.9	45.04	1.26
**Histamine**	32.7	69.7	1.62	20.8	40.25	1.41

**Table 5:** Topological parameters for bonds of interacting atoms of the three ratios of MIPs.

Hydrogen bond	*ρ* (a.u)	*▽*^2^_ρ_ (a.u)	*V*_BCP_ (a.u)	*G*_BCP_ (a.u)	*H*_BCP_ (a.u)	*E*_BCP_ (kJ/mol)	Length (Ǻ)
			**Ratio**	**1:3**			
N_1_–H_2_–O_63_	0.0102	0.0397	–0.00807	0.0090	0.000936	–10.593	3.077
N_1_–H_65_–O_64_	0.0307	0.215	–0.0502	0.0519	0.00174	–65.920	2.734
N_27_–H_27_–O_53_	0.0194	0.106	–0.0209	0.0237	0.00275	–27.455	3.002
O_51_–H_52_–O_39_	0.0251	0.1861	–0.0382	0.0423	0.00417	–50.117	2.707
O_40_–H_41_–N_27_	0.0383	0.294	–0.0722	0.0729	0.000670	–94.848	2.632
			**Ratio**	**1:4**			
N_10_–H_40_–O_21_	0.0195	0.106	–0.0210	0.0237	0.00276	–27.552	3.000
O_20_–H_22_–O_15_	0.0252	0.186	–0.0382	0.0424	0.00418	–50.150	2.706
O_64_–H_65_–N_10_	0.0385	0.296	–0.0729	0.0735	0.00610	–95.657	2.629
O_62_–H_63_–N_1_	0.0389	0.297	–0.0734	0.0739	0.000448	–96.424	2.628
O_76_–H_77_–O_61_	0.0252	0.187	–0.0385	0.0427	0.00418	–50.549	2.707
N_1_–H_24_–O_75_	0.0202	0.113	–0.0224	0.0253	0.00288	–29.476	2.978
			**Ratio**	**1:5**			
N_10_–H_40_–O_21_	0.0195	0.106	–0.0210	0.0238	0.00276	–27.590	3.001
O_15_–O_20_-H_22_	0.0251	0.186	–0.0381	0.0423	0.00417	–50.0195	2.708
O_64_–H_65_–N_10_	0.0386	0.297	–0.0731	0.0737	0.000606	–95.969	2.629
O_62_–H_63_–N_1_	0.0379	0.287	–0.0704	0.0711	0.000634	–92.489	2.639
O_76_–H_77_–O_61_	0.0253	0.188	–0.0386	0.0429	0.00419	–50.763	2.705
N_1_–H_24_–O_75_	0.0203	0.113	–0.0226	0.0255	0.00288	–29.661	2.975
O_88_–H_89_–N_5_	0.0160	0.212	–0.0503	0.0517	0.00136	–66.0895	2.742

**Table 6:** This Energy decomposition of SPD-MAA complex at different ratios. (unit: kJ/mol).

Ratio 1:3	Electrostatic	Repulsion	Dispersion
SPD-MAA	−54.84	64.57	−49.02
MAA-MAA	−23.62	13.94	−12.18
**total**	−78.46	78.51	−61.20
**Ratio 1:4**	**Electrostatic**	**Repulsion**	**Dispersion**
SPD-MAA	−68.67	81.15	−60.83
MAA-MAA	−46.60	−27.92	−23.91
**total**	−115.27	109.07	−84.78
**Ratio 1:5**	**Electrostatic**	**Repulsion**	**Dispersion**
SPD-MAA	−77.6	106.94	−89.28
MAA-MAA	−46.97	27.91	−24.59
**total**	−124.57	134.85	−113.87

**Table 7:** The energy of three amine MAA complex.

Compounds	Molecular Structure	Complex Energy (1:4 for MAA)
Spermidine	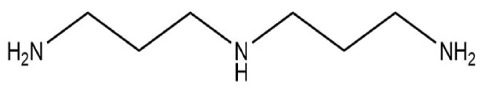	–1668.207 a.u
Tyramine	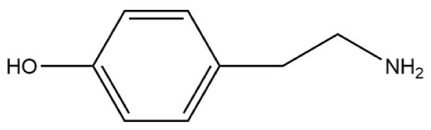	–1627.883 a.u
Histamine	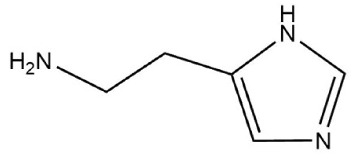	–1586.008 a.u
